# Establishing the Intergenerational Community Health Collaborative: An Innovative Partnership Model

**DOI:** 10.1093/geroni/igaf122.879

**Published:** 2025-12-31

**Authors:** Grace Savard, Maya Koffski, Azylen Lunak, Fyzeen Ahmad

**Affiliations:** University of Minnesota Twin Cities, Minneapolis, Minnesota, United States; University of Minnesota School of Public Health, Minneapolis, Minnesota, United States; University of Minnesota School of Public Health, Minneapolis, Minnesota, United States; University of Minnesota Department of Neuroscience, Minneapolis, Minnesota, United States

## Abstract

Established in Fall 2023, the Intergenerational Community Health Collaborative (ICHC) is a partnership initiative between the University of Minnesota’s Aging Studies Interdisciplinary Group—a GSA Student Chapter—and the Pillars of Prospect Park, a residential community for older adults located in Minneapolis, MN. The group meets monthly and includes over 35 members—University of Minnesota students and Pillars residents alike. Together, we explore Minnesota’s most pressing societal issues through an intergenerational lens. Grounded in shared leadership between students and residents, we cultivate curiosity and engagement through discussions, hosting expert speakers, and participating in civic advocacy efforts. Members collectively brainstorm and vote on focus areas at the beginning of each semester, and a planning committee of students and residents coordinates meetings and activities throughout the academic year. Over the past two years, we have focused on healthy aging initiatives, food insecurity in the Twin Cities, bridging divides through constructive dialogue, and protection from scams and fraud. This group uniquely positions members to develop intergenerational relationships and share lived experiences while participating in practical learning. In an effort to inform other GSA Student Chapters who may be interested in enacting a program similar to ICHC, this presentation will feature lessons learned about shared intergenerational leadership, tools utilized to sustain engagement from students and residents, and specific implementation challenges to consider. Finally, we will highlight future directions and goals of ICHC, emphasizing the potential for innovative intergenerational partnership and elevated community impact.

